# Rapid improvement of a complex migrainous episode with sodium valproate in a patient with CADASIL

**DOI:** 10.1007/s10194-011-0400-y

**Published:** 2011-11-08

**Authors:** Mika H. Martikainen, Susanna Roine

**Affiliations:** Department of Neurology, Turku University Hospital, Kiinamyllynkatu 4-8, 20521 Turku, Finland

**Keywords:** Aphasia, CADASIL, Migraine, Seizures, Sodium valproate

## Abstract

Cerebral autosomal dominant arteriopathy with subcortical infarcts and leukoencephalopathy (CADASIL) is an inherited disease of small arteries caused by mutations in the *Notch3* gene. Complex migrainous episodes, such as acute confusional migraine, status migrainosus with persisting aura, and “CADASIL coma” have been described in patients with CADASIL. However, there are few descriptions of effective treatment of such episodes. We describe a 44-year-old male with CADASIL, who presented with sudden-onset aphasia and decreased responsiveness after prolonged, severe migraine attack. Subsequently, the patient had two generalized seizures. A subtle status epilepticus was suspected because of drowsiness and seizures, and intravenous sodium valproate medication was initiated. EEG recording showed left hemispheric attenuation but no spike discharges, thus not confirming epileptic mechanism. The clinical status of the patient improved markedly after the initiation of valproate. The patient started speaking again; drowsiness and headache subsided. In repeated EEG recording, the left hemispheric attenuation disappeared. Diffusion weighted MR imaging showed no signs of recent ischemic events. The patient recovered fully from the episode with no further seizures. We suggest that CADASIL patients with acute complex migrainous episodes may benefit from intravenous sodium valproate.

## Introduction

Cerebral autosomal dominant arteriopathy with subcortical infarcts and leukoencephalopathy (CADASIL) is an inherited disease of small arteries caused by mutations in the *Notch3* gene [1, 2]. Complex migrainous manifestations, such as acute confusional migraine (ACM) [3], status migrainosus with persisting aura [4] and a more severe manifestation, the so-called “CADASIL coma” [5], have been described in patients with CADASIL. However, there are few reports on efficacious treatment of such episodes. Some patients with “CADASIL coma” have been treated with intravenous (i.v.) phenytoin [5]. Sodium valproate is used as both preventive and acute treatment of migraine. We report first successful treatment of an acute complex migrainous episode in a patient with CADASIL using i.v. valproate.

## Case report

We describe a 44-year-old male with CADASIL, a member of an identified CADASIL family. He had a confirmed R133C *Notch3* mutation. The patient also had type 2 diabetes mellitus, hypercholesterolemia, obesity, and slightly elevated blood pressure. Regular medications included acetylsalicylic acid, bisoprolol, simvastatin, metformin, glimepiride, and escitalopram. He had hemiplegic migraine (ICHD-II classification: 6.7.1; headache attributed to CADASIL. As to the ICHD-II classification of headache in CADASIL, a revision has recently been suggested [6]). Typical aura symptoms were right hemiparesis, confusion, and aphasia, and the duration of the migraine attacks was less than 1 day. There was no history of symptoms suggestive of epilepsy.

**Fig. 1:**
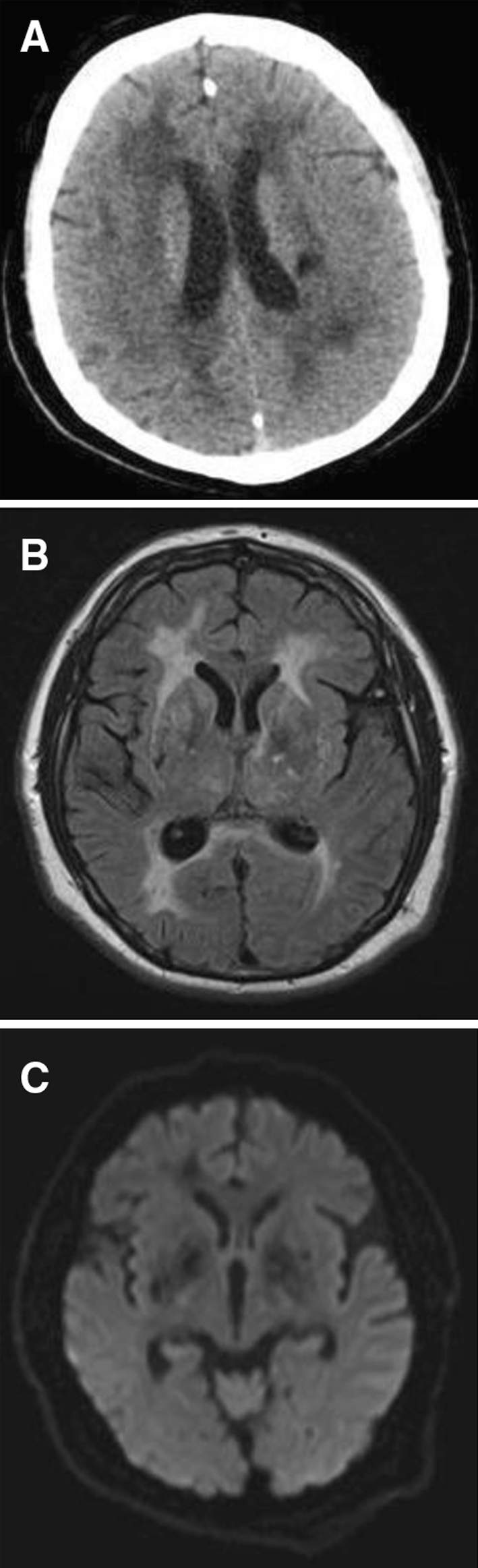
**a** Confluent symmetric leukoaraiosis typical of CADASIL on admission head CT. **b** Fluid-attenuated inversion recovery (FLAIR) series in brain MR imaging showed old basal ganglia infarctions in addition to extensive white matter hyperintensities. **c** Diffusion tensor MR imaging showed no signs of acute ischemia

The patient presented with sudden aphasia after prolonged, severe migraine attack with onset 3 days earlier. Initial clinical investigation showed global aphasia. Slight right hemiparesis, which was an old finding, was noticed. Flexor plantar responses were observed. The patient was conscious but responsiveness was decreased. He was not agitated. Body temperature, blood pressure and basic laboratory investigations (including plasma sodium, potassium, calcium, and free thyroxine) were normal. Plasma glucose level was 8.1 mmol/l. Arterial blood gases, ketone bodies, or magnesium or phosphate levels were not investigated. Autoimmune thyroid studies were not performed. There were no signs of meningeal irritation, and lumbar puncture was not performed. The patient and his family were known in the hospital, and nothing suggested intoxication or substance abuse. Thus no toxicology screen was performed. Brain CT (Fig. 1a) on admission to hospital showed symmetric leukoaraiosis characteristic of CADASIL and symmetric basal ganglia calcifications, but no new findings that would explain the acute symptoms. Perfusion CT imaging was not performed.

Initially, the patient was thought to have suffered an ischemic stroke, and he was admitted to neurological ward. He remained conscious, but somewhat drowsy and aphasic. Due to the patient’s aphasia, the persistence of headache was difficult to confirm. On the second day in hospital he had two brief generalized seizures. Because of drowsiness and seizures, a subtle status epilepticus was suspected and i.v. sodium valproate medication was initiated (first a loading dose of 800 mg i.v., the following day total 1,600 mg i.v. in three doses, 1,200 mg i.v. in two doses daily thereafter). Short-time EEG recording performed on the same day (before the initiation of valproate) showed left hemispheric attenuation, but no spike discharges or epileptic paroxysmal rhythmic activity. The clinical status of the patient improved markedly after the initiation of valproate. The patient started speaking again, he became responsive and drowsiness subsided. There were no further seizures. In a repeated EEG recording the following day, the left hemispheric attenuation disappeared. Brain MR imaging (Fig. 1b, c) on third day in hospital showed symmetric white matter ischemic lesions and basal ganglia calcifications in accordance with previous imaging findings. Diffusion weighted MR imaging showed no signs of recent ischemic events. MR angiography was normal. Perfusion MR imaging, PET, or SPECT imaging were not performed. The patient recovered fully from the episode with no further seizures in the follow-up. He has not had similar, severe migrainous episodes since. He has continued on a regular peroral valproate medication, 600 mg twice daily.

## Discussion

There seems to be some clinical continuity or overlap in several complex migrainous conditions described in CADASIL patients. In ACM, the symptoms of prolonged migraine with aura, impaired language functions and decreased responsiveness, or confusion and agitation are common [3]. A previously reported CADASIL patient with status migrainosus had several clinical neurologic deficits, including psychomotor slowness, speech difficulties, and a generalized seizure [4]. Among the six patients with “CADASIL coma” described in a previous work [5], the episodes typically started with migraine attacks, other common features being confusion, drowsiness, and other neurologic deficits. Four of these patients had seizures, and three had been treated with i.v. phenytoin medication. A previous CADASIL patient with non-convulsive status epilepticus (NCSE) presented with psychomotor slowing, confusion, left-sided neglect, and hemianopia [7]. The symptoms of the patient were also precipitated by a prolonged, severe migraine, and she was treated with i.v. phenytoin. There are, however, to our knowledge no previous reports on patients with CADASIL and migrainous conditions or seizures treated with i.v. valproate.

Sodium valproate has been used extensively in migraine prophylaxis, but its role in acute treatment of migraine is less established. However, in a recent report [8] i.v. sodium valproate was found to be effective in acute migraine treatment. Valproate has several mechanisms of action that explain its anti-migraine effects, including the increase of γ-aminobutyric acid (GABA) levels in the brain and the reduction of firing rates of serotonergic cells in dorsal raphe [8]. Interestingly, findings of another recent study [9] suggested that valproate might have beneficial effects in CADASIL via cytoprotective effects on vascular smooth muscle cells.

The clinical features of our patient are compatible with a complex migrainous episode in CADASIL. The presentation of severe aura symptoms (aphasia) after the onset of migrainous headache is unusual, but this may be the case in CADASIL. Typically these episodes present after a prolonged migraine with symptoms of decreased responsiveness, confusion, language difficulties, focal neurologic deficits such as hemianopia or hemiparesis, and sometimes seizures. The patient showed definite clinical response to i.v. sodium valproate medication. We suggest that in CADASIL patients with acute, complex migrainous episodes, i.v. sodium valproate may be a good therapeutic option.

*Ethical statement* High standard of ethics according to the WMA Declaration of Helsinki was applied in all investigations and clinical work described in this manuscript.
